# Place de la laparoscopie dans la prise en charge des anomalies de différenciation sexuelle: à propos de 4 cas

**DOI:** 10.11604/pamj.2016.23.167.8810

**Published:** 2016-04-07

**Authors:** Aissam Goultaiene, Khalid Elmortaji, Reda Sentissi, Amine Moataz, Redouane Rabii, Rachid Aboutaib, Mohammed Dakir, Adil Debbagh, Fethi Meziane

**Affiliations:** 1 Service d'Urologie, CHU Ibn Rochd, Casablanca, Maroc

**Keywords:** Ambigüité sexuelle, différenciation sexuelle, laparoscopie, Laparoscopy, sexual ambiguity, sex development

## Abstract

Les troubles de la différenciation sexuelle sont à l'origine d'une discordance entre le sexe proprement dit (phénotypique) et le sexe génétique (génotypique) ce qui pose un problème de détermination du sexe. Dans les pays de faible niveau socio-économique où le diagnostic anténatal est souvent absent et les plateaux techniques insuffisants, la prise en charge médico-chirurgicale est difficile. Le but de ce travail est de préciser la place de la laparoscopie dans la prise en charge de l'ambiguïté sexuelle à travers l'observation de 4 cas et une revue de la littérature.

## Abstract (en)

Disorders of sex differentiation cause a discrepancy between sex itself (phenotype) and genetic sex (genotype) which poses a problem in sex determination. In lower socioeconomic level countries where prenatal diagnosis is often absent and technical equipments are inadequate, medical and surgical management is difficult. The aim of this study is to clarify the role of laparoscopy in the management of sexual ambiguity through observation of 4 cases and review of the literature.

## Introduction

Les troubles de la différenciation sexuelle sont des états intersexués rares, elle touche environ un nouveau-né sur 4500 naissances [1] et se caractérisant par un mélange en proportions variables de caractères sexuels masculins et féminins. Selon la nouvelle nomenclature [2] on ne parle plus d'ambiguïté sexuelle mais d'anomalies de la différenciation sexuelle (ADS). Cette classification est basée sur le caryotype. Les anomalies de la différenciation sexuelle (disorders of sex development DSD) résultent d'une masculinisation insuffisante d'un embryon génétiquement masculin (46 XY, DSD) ou d'une virilisation excessive d'un embryon féminin (46 XX, DSD), où plusieurs facteurs génétiques et hormonaux sont impliqués [3]. L'examen clinique méthodique et l'investigation hormonale, radiologique, moléculaire et génétique conduisent au diagnostic d'une ambiguïté génitale chez le nouveau-né. Sa prise en charge nécessite la présence d'une équipe multidisciplinaire expérimentée. Nous rapportons l'observation de 4 cas ambiguïté sexuelle, traités par cœlioscopie.

## Patient et observation

**Figure 1:**
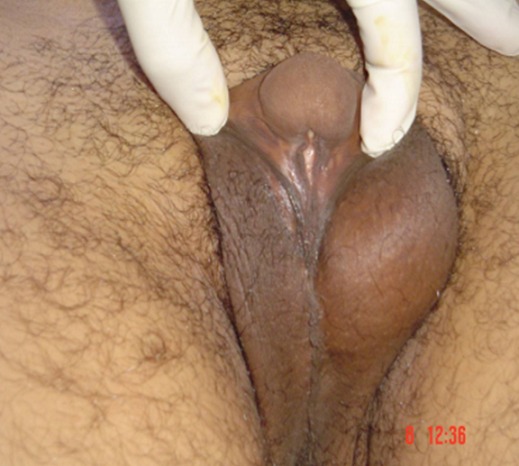
Micropénis vulviforme, hypospade; scrotum bifide, bourses vides, pilosité pubienne de type masculine

**Figure 2:**
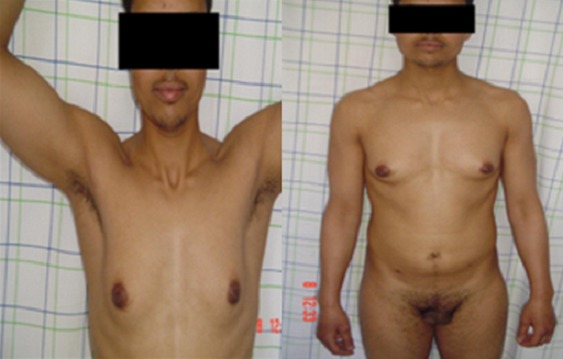
Pilosité pubienne de type masculin, pilosité axillaire, barbe, moustache et gynécomastie

**Figure 3:**
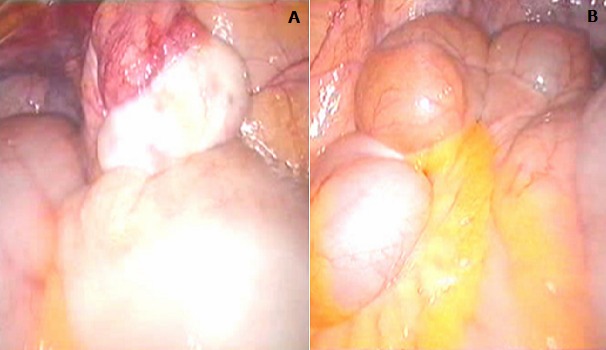
A) Gonade droite atrophique de 1 cm coiffée par un pavillon; B) gonade gauche au niveau de l'orifice inguinal profound

**Figure 4:**
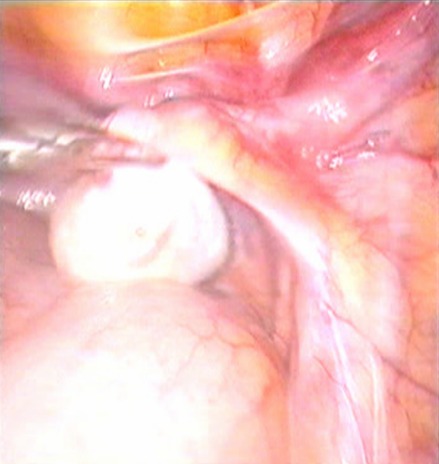
Ablation laparoscopique de la gonade droite

**Figure 5:**
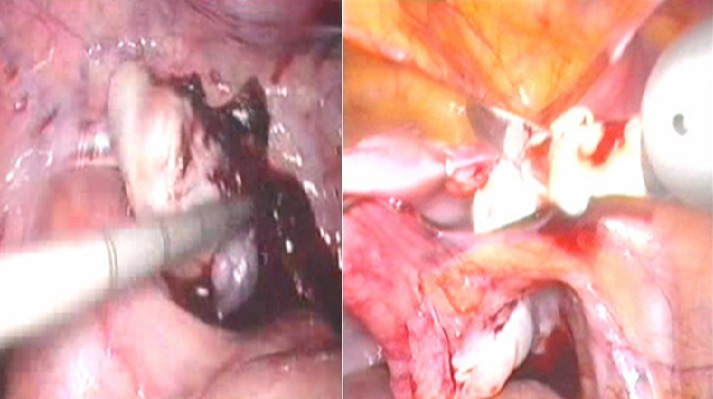
Abaissement laparoscopique en intrascrotal de la gonade gauche

**Observation 1:** Il s'agit d'un patient âgé de 28 ans, qui consulte pour des testicules non palpables. L'interrogatoire ne retrouve pas d'antécédents pathologiques particuliers, et l'examen clinique retrouve un micropénis (3 cm) vulviforme, hypospade, un scrotum bifide, une bourse vide, la présence d'une pilosité pubienne et axillaire, la présence de barbe, de moustache et d'une gynécomastie (Figure 1, Figure 2). Un bilan hormonal a révélé une testostéronèmie normale, le taux de la FSH, LH et de la 17-OH progestérone étaient normaux. Le caryotype a été réalisé et a révélé 46 XX. Un scanner abdomino-pelvien a montré un aspect normal des surrénales sans autres anomalies. Devant ce tableau, une exploration cœlioscopique a retrouvé une gonade droite atrophique de 1cm coiffée par un pavillon et une gonade gauche au niveau de l'orifice inguinal profond (Figure 3 A, B, C). On a procédé à l'ablation de la gonade droite (Figure 4) et à l'abaissement en intrascrotal de la gonade gauche qui a l'aspect d'un testicule normal (Figure 5). L’étude anatomopathologique de la gonade droite a mis en évidence la coexistence de deux tissus : Ovarien et testiculaire en faveur d'un hermaphrodisme vrai. Gestes ultérieurs prévus : prise en charge psychiatrique, cure de l'hypospadias, biopsie de la gonade gauche, réduction mammaire par un traitement médical et traitement hormonal substitutif si la biopsie de la gonade gauche révèle un ovotestis ou un ovaire.

**Figure 6:**
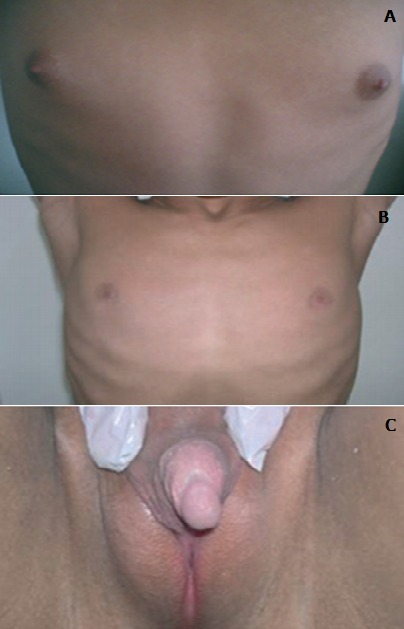
A) Développement mammaire; B) Absence de pilosité axillaire, C) hypertrophie clitoridienne

**Observation 2:** Il s'agit d'une patiente âgée de 16 ans, deuxième d'une fratrie de deux, sans antécédents pathologiques particuliers, consulte pour aménorrhée primaire. L'examen gynécologique ne retrouve pas de développement mammaire, il n'y a pas de pilosité axillaire ni pubienne. L'examen de la sphère génitale retrouve un clitoris hypertrophié, un vagin peu profond (Figure 6 A, B, C). Le toucher rectal ne retrouve pas d'utérus palpable. Un bilan hormonal a révélé un taux plasmatique élevé de testostérone et de delta 4 androstènedione à 9 ng/ml et 2,8 ng/ml respectivement. Par ailleurs, le dosage sanguin a révélé: FSH à 7 mUI/ml, LH à 17,6mUI/ml et œstradiol à 39 pg/ml. L’étude cytogénétique a montré un caryotype de type masculin XY. Une échographie pelvienne a été demandée et qui a montré deux formations tissulaires, ovalaires, bien limitées hypoéchogènes homogènes situées en dedans des vaisseaux iliaques. L'IRM pelvienne a montré la présence en dedans des vaisseaux iliaques de deux formations ovalaires bien limitées, iso signal aux parties molles en T1, hyper signal T2, entourées de couronnes en hypo signal et se rehaussant de façon homogène après injection de gadolinium. La patiente a bénéficiée d'une orchidectomie bilatérale sous cœlioscopie et un traitement hormonal à base d'oestroprogestatif, ayant entraîné une augmentation progressive du volume mammaire.

**Figure 7:**
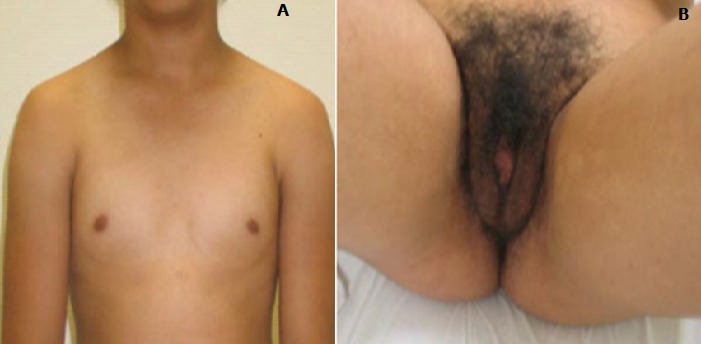
A) pas de développement mammaire; B) hirsutisme

**Observation 3:** Il s'agit d'une patiente âgée de 16 ans, deuxième d'une fratrie de deux, sans antécédents pathologiques particuliers qui consulte pour virilisation avec aménorrhée primaire. L'examen clinique trouve une hypertrophie clitoridienne avec bourrelets striés et gonades palpables aux niveaux des deux plis inguinaux (Figure 7 A, B). La patiente a était classé Prader stade 4 Un caryotype demandé était de type masculin 46XY. Un bilan hormonal a révélé: Testostérone à 6,8 ng/mL (le normal chez le sujet sexe masculin est situé entre 3,5-8,5 ng/mL). Dihydrotestostérone à 0.17ng/mL FSH à 13,3 UI/L, LH à 4,3UI/L (normaux). La génitographie demandée n'a pas montrée de vestiges müllériens. Une échographie pelvienne faite a montrée la présence de deux formations tissulaires, ovalaires, bien limitées hypoéchogènes homogènes situées au niveau des orifices inguinaux internes avec l'absence de vestiges müllériens. Un IRM pelvienne demandé a montré la présence au niveau des orifices inguinaux internes de deux formations ovalaires bien limitées, iso signal aux parties molles en T1, hyper signal T2, entourées de couronnes en hypo signal et se rehaussant de façon homogène après injection de gadolinium. La patiente a bénéficié d'une laparoscopie à but diagnostique qui n'a pas objectivée de vestiges müllériens, mais elle a révélée des gonades au niveau des orifices inguinaux internes. Donc un abaissement cœlioscopique des gonades au niveau du scrotum été fait. On a retenu comme diagnostic étiologique un déficit en 5 alpha réductase.

**Observation 4:** Il s'agit d'un patient âgé 14 ans consulte pour anomalie de développement sexuelle sans antécédents pathologiques notables. L'examen clinique trouve un bourgeon génital de 40 mm/20 mm bourrelets de type féminin et pas de gonades palpables, Un seul orifice au niveau de la région vulvaire. Le patient était classé Prader stade 3. Un caryotype demandé était de type masculin 46 XY. Le bilan hormonal a montré le delta 4 androstènedione à 1,15 nmol/ml(N), Dihydrotestostérone à 0.19 nmol/ml (N), Testostérone: 0.30 nmol/ml (N). L’échographie pelvienne a montrée une formation anéchogène rétrovesicale évoquant une cavité vaginale avec gonade gauche au niveau du pli inguinal et gonade droit et utérus non visualisés. La génitographie demandée a montrée un urètre court type masculin,cavité vaginal bien développée. Le patient a bénéficié d'une laparoscopie à visé diagnostique qui a objectivée des vestiges müllériens, testicule gauche au niveau inguinal hypotrophique avec indépendance épydidimo-testiculaire totale avec insertion de la trompe gauche sur le testicule gauche. Une section ligature de la trompe gauche avec abaissement du testicule gauche en intra scrotal été faite. On a retenu comme diagnostic une dysgénésie gonadique partielle avec respect de l'orientation sexuelle du patient comme sexe masculin. Le patient fut programmé pour cure de l'hypospadias avec substitution par testostérone et surveillance clinique et échographique (risque de dégénérescence gonadique).

## Discussion

L'ambiguïté sexuelle est définie comme un aspect non ou mal différencié des organes génitaux externes ou à une discordance entre les organes génitaux internes et l'aspect des organes génitaux externes; elle reste une pathologie complexe et rare avec une prévalence de 0,1 à 2% selon certains auteurs [4]. Sa prise en charge est basée sur l’élaboration d'une stratégie thérapeutique bien définie. L’échographie et l'IRM sont considérées comme des examens clés dans le diagnostic paraclinique; Néanmoins durant les dernières décennies, la laparoscopie occupe de plus en plus une place prépondérante dans le diagnostic et la prise en charge thérapeutique [5]. Elle permet une excellente visualisation des gonades par rapport à l'imagerie qui reste limité dans certains cas difficiles [6, 7], notamment, en cas de testicules non palpables, l'exploration laparoscopique reste plus sensible et plus fiable dans l’évaluation des organes génitaux internes chez les patients qui présentent des désordres de développement sexuel [8]. Quant au traitement; l'attitude conservatrice et la correction des désordres anatomiques sous cœlioscopie constituent les modalités de référence [9, 10], avec un suivi hormonal obligatoire chez ces patients qui ont bénéficié de cette attitude et une surveillance rigoureuse à long terme vu le risque de dégénérescence [5]. En outre, l'approche laparoscopique présente encore plus d'avantages dans la prise en charge des désordres du développement sexuel en terme de complications per et post-opératoires ainsi que sur le préjudice esthétique [5] qui reste un facteur important impliqué dans la constitution de la psychologie de ces patients.

Dans notre travail, on a procédé à une conservation gonadique avec abaissement en intra-scrotal chez trois patients et une gonadectomie bilatérale chez un seul patient dont l’étude anatomo-pathologique a objectivé la présence de la coexistence de deux tissus: Ovarien et testiculaire. Tous nos patients ont bénéficié d'une prise en charge psychiatrique avec une réintégration sociale.

## Conclusion

Les anomalies de différenciation sexuelle sont une entité rare, dont leur prise en charge médico-chirurgicale est difficile. L'approche laparoscopique occupe de plus en plus une place importante dans cette prise en charge. Elle permet une exploration diagnostique plus fiable et l’élaboration de l'attitude stratégique adéquate dans ce type d'anomalies avec un minimum de complications et de préjudice esthétique.
